# The Predictive Value of Adrenomedullin for Development of Severe Sepsis and Septic Shock in Emergency Department

**DOI:** 10.1155/2013/960101

**Published:** 2013-08-04

**Authors:** Yun-Xia Chen, Chun-Sheng Li

**Affiliations:** Emergency Department of Beijing Chao-Yang Hospital, Affiliated to Capital Medical University, Chaoyang District, Beijing 100020, China

## Abstract

*Objective*. The aim of the study was to assess adrenomedullin (AM) as a predictor for development of severe sepsis and septic shock in emergency department (ED). *Method*. From December 2011 to October 2012, 372 consecutive septic patients admitted to ED were enrolled. AM was examined in every patient. All patients were followed up for 3 days. The outcome variable was development of severe sepsis or septic shock. The predictive ability of AM was evaluated by binary logistic regression analysis and receiver operating characteristic (ROC) curve. *Result*. On admission, the differences of AM among patients with different comorbidities, infections, and culture results were not significant. AM level was higher in patients who progressed than in who did not (41.63 ± 6.55 versus 31.31 ± 7.71 ng/L, *P* < 0.001). AM was the only independent predictor of outcome. The area under ROC curve of AM was 0.847. With a cutoff value of 41.24 ng/L, the sensitivity was 67.6%, the specificity was 90.0%, the positive predictive value was 61.5%, the negative predictive value was 92.2%, the positive likelihood ratio was 6.78, and the negative likelihood ratio was 0.36. *Conclusion*. Adrenomedullin is valuable for predicting development of severe sepsis and septic shock in ED.

## 1. Introduction

Despite advances in the resuscitation of sepsis, the incidence of severe sepsis and septic shock has increased significantly in recent decades. Sepsis, septic shock, and the ensuing multiple organ failure continue to be the most common causes of death in critically ill patients admitted to the emergency department (ED) and intensive care unit (ICU). The mortality is about 20~50% in severe sepsis and 40~80% in septic shock [1]. Early diagnosis and appropriate classification play a crucial role in treatment decisions for sepsis. Delay in diagnosis means delay in intervention in the early periods of sepsis when appropriate management strategies can be instituted before irreversible organ damage occurs.

Adrenomedullin (AM) is a 52-amino-acid peptide that was first isolated from pheochromocytomas [2]. Subsequent studies have shown that AM is produced and secreted by many mammalian tissues and endothelial cells. Stimuli for AM synthesis and secretion include angiotensin II, endothelin-1, hypoxia, oxidative stress, and inflammatory cytokines such as tumor necrosis factor-*α* and interleukin-1*β*. AM possesses anti-inflammatory, bactericidal, positive inotropic, and perhaps most importantly, vasodilatory activities [3]. Several clinical studies have demonstrated that AM increases significantly in septic patients and is correlated with disease severity. In patients with septic shock, AM peptide levels are 25–30-fold higher than in normal individuals [4–7]. But researches assessing the ability of AM for predicting the organ dysfunction and shock in septic patients have not been reported. The present study aimed to evaluate the ability of AM in septic patients on ED admission as a predictor of organ dysfunction and shock.

## 2. Materials and Methods

### 2.1. Patients

**Figure 1:**
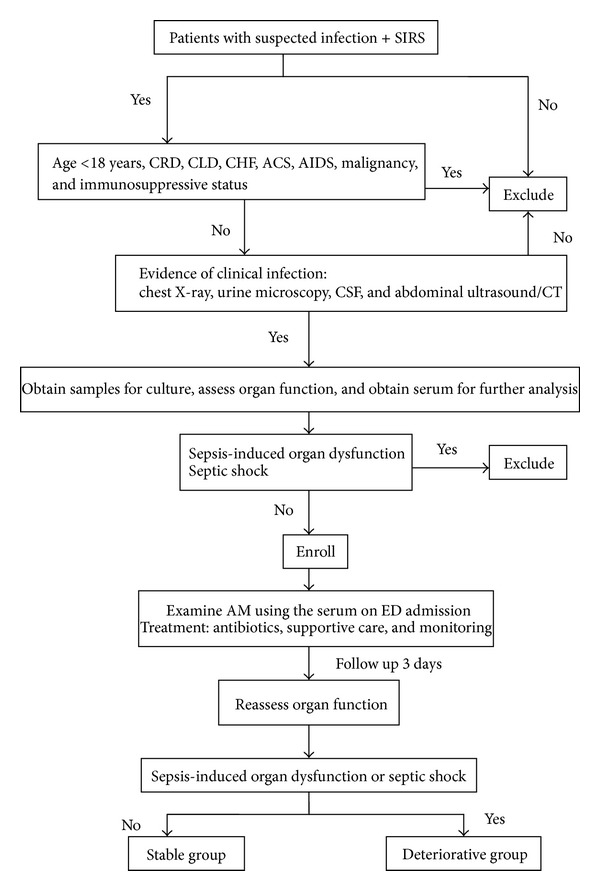
The flow diagram of patients enrollment and grouping (CRD: chronic renal disease; CLD: chronic liver disease; CHF: chronic heart failure; ACS: acute coronary syndrome; AIDS: acquired immune deficiency syndrome).

The single-center observational study was conducted in the ED of Beijing Chao-Yang Hospital, which is an urban university tertiary hospital with approximately 250,000 ED visitors per year. From December 2011 to October 2012, 372 consecutive patients who fulfilled the sepsis criteria defined by American College of Chest Physicians/Society of Critical Care Medicine (ACCP/SCCM) were enrolled [8]. The exclusion criteria were as follows: less than 18 years old; terminal stage of disease (malignant cancer of any type, acquired immune deficiency syndrome (AIDS), and end-stage renal or liver disease); immunosuppressive status; conditions that influence AM level (chronic heart failure; acute coronary syndrome); and patients who declined to participate the study by themselves or their relatives. The study was approved by the local ethics committee. Written informed consent was obtained from every patient. The procedure of patients' enrollment and grouping was shown as in Figure 1.

### 2.2. Outcome Variables of the Study

Patients were followed up for 3 days. Organ functions were reassessed by the end of 3-day followup or when the clinical status deteriorated. Development of severe sepsis or septic shock during follow-up was considered as the primary outcome. If patients already had organ dysfunction due to comorbidity, dysfunction of another organ induced by sepsis at the followup period was defined as severe sepsis.

Severe sepsis was defined as the presence of sepsis and at least one of the following manifestations of organ dysfunction: sepsis-induced hypotension (mean artery pressure < 65 mmHg); lactate greater than the upper limits of normal laboratory results (>2.5 mmol/L in present study); creatinine > 176.8 umol/L; urine output < 0.5 mL/kg/h for 2 hours, despite adequate fluid resuscitation; acute lung injury (ALI) with PaO_2_/FiO_2_ <250 in the absence of pneumonia as infection source; ALI with PaO_2_/FiO_2_ <200 in the presence of pneumonia as infection source; bilirubin > 34.2 umol/L; platelet count < 100 × 10^9^/L; International Normalized Ratio (INR) > 1.5.

Septic shock was defined as the presence of sepsis accompanied by a sustained arterial hypotension (systolic arterial pressure < 90 mmHg; mean arterial pressure < 60 mmHg, or a reduction in systolic blood pressure of more than 40 mmHg from baseline) despite adequate volume resuscitation, in the absence of other causes of hypotension.

### 2.3. Data Collection

Basic information of patient including age, gender, and comorbidity were recorded at enrollment. Investigations associated with clinical sign of infection were done in order to identify the infection source, including X-ray, ultrasound, CT, urine, and other body fluid test. Culture of blood, sputum, urine, and other samples was done for sake of identifying pathogen and guiding therapy. Acute Physiology and Chronic Health Evaluation (APACHE) II score and Sequential Organ Failure Assessment (SOFA) score were calculated in every patient using the data at enrollment.

### 2.4. Measurement Methods

Venous blood samples were obtained at the time of ED admission. Serum was separated by centrifugation at 3000 rpm for 5 min and stored at −80°C until assayed. AM was analyzed using enzyme-linked immunosorbent assay (ELISA) by a professional analyzer corporation. AM was detected by double antibody sandwich method. The ELISA assay included a purified human anti-AM antibody as the capture antibody, a horseradish peroxidase-(HRP-) conjugated human antibody against AM, perborate/3, 3, 5, 5-tetramethylbenzidine as the substrate.

### 2.5. Statistical Analysis

All data were analyzed by SPSS version 13.0 (SPSS Inc., Chicago, IL, USA). Normal distributed data were expressed as mean ± standard deviation and compared by Kruskal-Wallis one-way analysis of variance. Skewed distributed data were expressed as median and quartiles and analyzed by Mann-Whitney *U* test. Comparison of frequencies was done using the *χ* ^2^ test. Receiver operating characteristic (ROC) curve was constructed, and the area under the ROC curve (AUC) was determined to assess the predictive value of AM. On the basis of optimal threshold determined by ROC curve, prognostic parameters (sensitivity, specificity, and positive and negative predictive values) were also calculated. Binary logistic regression analysis was used to determine the independent predictors of severe sepsis and septic shock. All statistical tests were two-tailed, and *P* < 0.05 was considered statistically significant.

## 3. Results

### 3.1. Patient Characteristics

As shown in Table 1, by the end of 3-day followup, there were 71 patients progressed to severe sepsis or septic shock (deteriorative group), others did not (stable group). The differences in age (72 versus 71, *P* = 0.825) and gender (64.8% versus 61.1%, *P* = 0.59) were not significant between groups. The incidences of major comorbidities were not significantly different between the two groups. The infection sites were not different between groups. The ratio of positive culture result was higher in deteriorative patients than in stable ones, but the difference was not significant (63.4% versus 50.8%, *P* = 0.064). Also, the APACHE II score and SOFA score were not different between the two groups.

### 3.2. AM Levels

#### 3.2.1. AM Levels in Patients with Different Comorbidities

**Figure 2:**
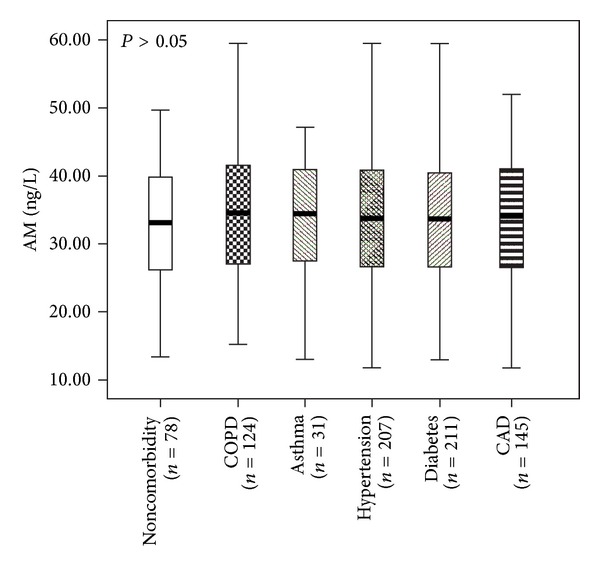
The mean levels of AM in patients with different comorbidities (COPD: chronic obstructive pulmonary disease; CAD: coronary arterial disease).

The mean level of AM was 34.26 ± 8.54 (ng/L) in patients with COPD, 33.84 ± 8.27 in asthma, 33.42 ± 8.71 in hypertension, 33.15 ± 8.47 in diabetes, 33.42 ± 8.74 in stable CAD without HF, and 33.18 ± 8.40 in patients without comorbidity. There were not differences among all patients (*P* = 0.912) and between each two groups (*P* > 0.05). The result was shown in Figure 2.

#### 3.2.2. AM Levels in Patients with Different Infection

**Figure 3:**
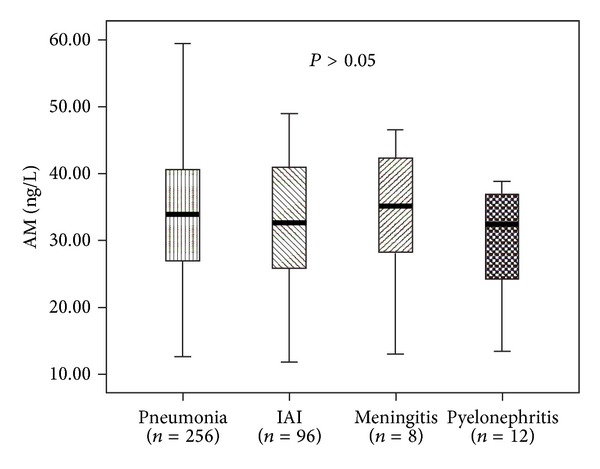
The mean levels of AM in patients with different infection (IAI: intraabdominal infection).

The mean levels of AM were 33.61 ± 8.29, 33.82 ± 10.89, 30.15 ± 7.89, and 32.75 ± 90.1 ng/L in patients with pneumonia, meningitis, pyelonephritis, and intraabdominal infection (IAI), respectively. The differences were not significant (Figure 3).

#### 3.2.3. AM Levels in Patients with Different Culture Result

**Figure 4:**
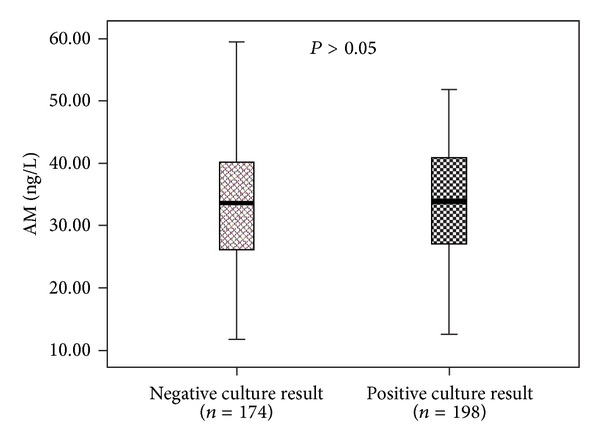
The mean levels of AM in patients with different culture result.

The difference of AM between patients with positive and negative culture results was not significant (33.39 ± 8.55 versus 33.16 ± 8.51, *P* = 0.796) (Figure 4).

#### 3.2.4. AM Levels in Patients with Different Outcome

**Figure 5:**
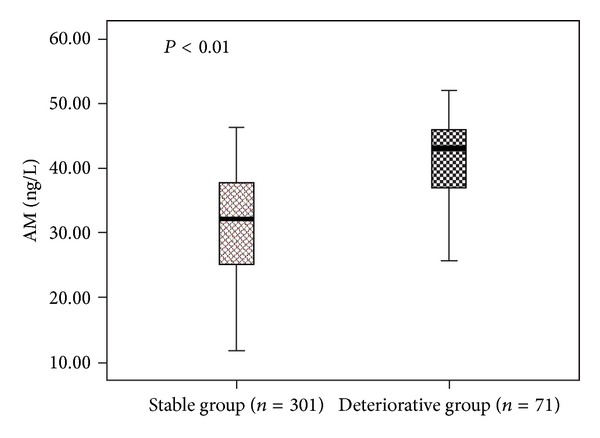
The mean levels of AM in patients developed severe sepsis or septic shock (deteriorative group) and who did not (stable group).

AM levels on ED admission were 41.63 ± 6.55 ng/L in patients who progressed to severe sepsis/septic shock and 31.31 ± 7.71 ng/L in patients who did not (*P* < 0.0001). The result was shown in Figure 5.

### 3.3. The Independent Predictor of Severe Sepsis and Septic Shock

We chose age, gender, comorbidity, infection site, APACHE II score, and SOFA score as the candidate variables of independent predictor for severe sepsis and septic shock along with AM. In a binary logistic regression analysis, AM was the only independent predictor of outcome. The result was shown in Table 2.

### 3.4. The Predictive Ability of AM

**Figure 6:**
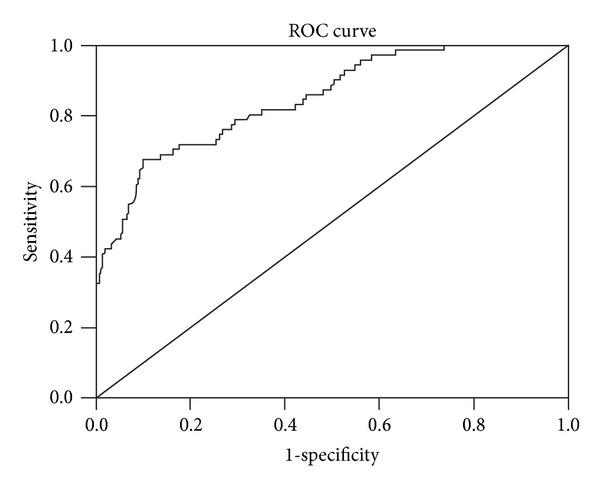
The ROC curve of AM predicting severe sepsis and septic shock.

The ROC curve of AM predicting severe sepsis and septic shock was shown in Figure 6. 

The area under ROC curve (AUC) was 0.847 (95% CI: 0.797–0.898, *P* = 0). With an AM cutoff value of 41.24 ng/L, the sensitivity of the test was 67.6%, the specificity was 90.0%, the positive predictive value (PPV) was 61.5%, the negative predictive value (NPV) was 92.2%, the positive likelihood ratio was 6.78, the negative likelihood ratio was 0.36, Youden's index was 0.58, and the predict accuracy was 85.8%. The incidence of severe sepsis and septic shock when AM above the cutoff value was 67.6%.

## 4. Discussion

The present study revealed that circulating AM was higher in the patients who developed severe sepsis or septic shock within 3 days of ED arrival than in who did not. Therefore, AM may be a valuable predictor of deterioration of septic patients in ED.

### 4.1. AM Level in Septic Patients

The present study revealed that AM levels in septic patients with different comorbidities were not statistically different. This result was not reported before. There was no difference in AM between septic patients who were healthy before and who were not. So it is reasonable to consider that AM was induced by sepsis and did not correlate to the previous health status. According to the result of our study, the infection sites had no influence on the AM level. Between patients with positive and negative culture results, AM level had no statistical difference too.

Many studies have demonstrated that AM is produced and secreted from various cells, including peripheral blood granulocytes, lymphocytes, monocytes, monocyte-derived macrophages, and fibroblasts; all of these are involved in the inflammatory process. AM is induced by various factors during sepsis, such as catecholamine, hypoxia, oxidative stress, inflammatory mediators, and cytokines [3]. Most of these factors are originated from the acute pathophysiological changes of sepsis and do not associate with comorbidities, infection sites, and pathogens.

### 4.2. The Predictive Value of AM

The present study found that AM level on ED admission was much higher in patients who progressed to severe sepsis or septic shock than in those were relatively stable, and it was the only independent predictor of deterioration. A clinical study demonstrated that AM increased in proportion to the severity of illness in septic patients and consumed that AM might serve as a useful marker for evaluating the severity of disease and as an early predictor of subsequent organ failure and outcome in septic shock [4]. But the study did not evaluate the predictive ability of AM for deterioration of sepsis. Several studies revealed that prohormone (proADM) or midregional part of the prohormone (MR-proADM) of AM was prognostic in septic patients [9–13]. These studies mainly enrolled ICU admission patients and contained relatively small sample size. The outcome variable of these studies was mortality. The applicability of the results in septic patients in ED was still indefinitely. Also, these studies did not assess the prognostic value of biomarkers in predicting the development of severe sepsis and septic shock.

As mentioned previously AM was induced by many factors during the progression of sepsis. The illness was more severe, the inducing factors were more, and the AM was higher. On the other hand, AM was secreted and cleaned out rapidly in circulation. AM increased 2~4 hours after the setup of animal septic model [14, 15]. And its half-life time in circulation was only 20 minutes [16]. AM is a more timely biomarker of illness severity compared with other biomarkers which need longer time to be secreted and cleaned out. The persistent high level of circulation AM suggests the continuing existence of inducing factors. Therefore, high level AM indicates the severity and the deteriorative probability of septic patients.

Score systems are perfect tools of assessing severity of illness because they contain many clinical and biochemistry variables. But the present study found that APACHE II score and SOFA score were not different between patients who progressed and who did not, and they were not the independent predictors of deterioration. These results may be due to the variables that contained in the score systems are not able to predict the progression of disease.

The present study revealed that the positive rate of culture result was higher in patients who progressed to severe sepsis or septic shock than in who did not. But the difference between the two cohorts was not statistically significant. The positive culture result was not an independent predictor of deterioration.

According to the result of the present study, the AUC of AM predicting severe sepsis and septic shock was 0.847, and the sensitivity was 67.6%, the specificity was 90.0%, the PPV was 61.5%, and the NPV was 92.2% as the cutoff value was 41.24 ng/L. Therefore, AM may be suitable to serve as an excluding criterion of deterioration. Since the incidence of severe sepsis and septic shock was 67.6% when AM was above the cutoff value in our cohort, and the outcome of severe sepsis and septic shock was extremely adverse, we suggest giving more intensive monitoring to patients whose AM exceed the cutoff value and further assessing the severity of illness combined with other methods in order to minimize the probability of deterioration and improve outcome.

## 5. Limitations

The present study was a single-centre research and contained a relatively small cohort. Well-designed, larger sample and multicentre clinical studies will be needed to further investigate the results.

## 6. Conclusions

The present study revealed that AM is a valuable predictor of development of severe sepsis and septic shock in ED.

## Figures and Tables

**Table 1:** Patient characteristics.

Variables	Deteriorative group	Stable group	*P*
*N*	71	301	
Age (years)	72 (60–78)	71 (59–78)	0.825
Male, *n* (%)	46 (64.8)	184 (61.1)	0.590
Comorbidities, *n* (%)			
COPD	26 (36.6)	98 (32.6)	0.576
Asthma	5 (7.0)	26 (8.6)	0.813
Hypertension	42 (59.2)	165 (54.8)	0.596
Diabetes	36 (50.7)	175 (58.1)	0.287
Stable CAD without HF	29 (40.8)	116 (38.5)	0.787
Noncomorbidity	16 (22.5)	62 (20.6)	0.746
Infection site, *n* (%)			
Pneumonia	48 (67.6)	208 (69.1)	0.887
IAI	18 (25.4)	78 (25.9)	1.000
Meningitis	3 (4.2)	5 (1.7)	0.181
Pyelonephritis	2 (2.8)	10 (3.3)	0.590
Positive culture result, *n* (%)	45 (63.4)	153 (50.8)	0.064
APACHE II score	14.14 ± 6.26	14.05 ± 6.34	0.834
SOFA score	3 (2–5)	3 (2–5)	0.237

ED: emergency department; COPD: chronic obstructive pulmonary disease; CAD: coronary arterial disease; HF: heart failure; IAI: intraabdominal infection; APACHE: acute physiology and chronic health evaluation; SOFA: Sequential Organ Failure Assessment.

**Table 2:** The independent predictor of severe sepsis and septic shock.

	B	S.E.	Wald	*P*	Exp(B)	95.0% C.I. for EXP(B)	
						Lower	Upper
AM	0.217	0.028	58.459	0.000	1.243	1.175	1.314
Gender	−0.156	0.327	0.228	0.633	0.855	0.450	1.625
Age	−0.008	0.011	0.497	0.481	0.992	0.972	1.014
SOFA	0.059	0.091	0.413	0.520	1.060	0.887	1.268
APACHE II	−0.015	0.034	0.185	0.667	0.985	0.921	1.054
Comorbidity	−0.164	0.403	0.166	0.684	0.849	0.385	1.871
Infection site	0.233	0.237	0.962	0.327	1.262	0.793	2.010
Positive culture	0.585	0.327	3.196	0.074	1.794	0.945	3.407
Constant	−9.330	1.539	36.756	0.000	0.000		

AM: adrenomedullin; SOFA: Sequential Organ Failure Assessment; APACHE: acute physiology and chronic health evaluation.
